# Elementary Principles of Electro-Therapeutics

**Published:** 1884-07

**Authors:** 


**Elementary Principles of Electro-Therapeutics. For the use of physicians and students. With 135 illustrations. By C. M. Haynes, M. D. Published by the McIntosh Galvanic and Faradic Co., Chicago, Ill. Price, $2.00.**

This is a book of nearly 500 pages, which, from the number of its illustrations of the McIntosh batteries and electrodes, we considered, at first glance, to be primarily intended as an advertisement of that enterprising firm. This doubtless was an object in its publication, but it is not offensively apparent. Indeed, the work contains a vast amount of useful information. The elementary principles of magnetism, Franklinism, Galvanism and Faradism are well given, and the foundation principles of the applications of electricity in medicine are well taught. For the physician who wants to get at the practical facts of medical electricity in the easiest way, we know of no work more desirable; and to those who do not make medical electricity a special study, it will serve as a most useful guide to the intelligent use of their batteries.

